# Mental health in adolescents displaced by the armed conflict: findings from the Colombian national mental health survey

**DOI:** 10.1186/s13034-020-00327-5

**Published:** 2020-05-19

**Authors:** Arturo Marroquín Rivera, Carlos Javier Rincón Rodríguez, Andrea Padilla-Muñoz, Carlos Gómez-Restrepo

**Affiliations:** 1 Department of Clinical Epidemiology and Biostatistics, School of Medicine, Pontificia Universidad Javeriana, Bogotá, Colombia; 2 Human Rights Research Group, Faculty of Law, Universidad del Rosario, Bogotá, Colombia; 3 Department of Clinical Epidemiology and Biostatistics, Department of Psychiatry and Mental Health, Hospital Universitario San Ignacio, School of Medicine, Pontificia Universidad Javeriana, Kra. 7 N. 40–62 2nd Floor, Bogotá, Colombia

**Keywords:** Adolescence, Conflict, Internal displacement, Mental health, Colombia

## Abstract

**Background:**

Colombia has one of the largest populations of internally displaced individuals by an armed conflict. However, there is no data demonstrating its effect on health, particularly in adolescents.

**Purpose:**

To describe the prevalence and associations of mental illness in the adolescent population displaced by violence in Colombia.

**Methods:**

We conducted a secondary analysis of the 2015 National Mental Health Survey (NMHS), which provides data of mental health issues (SRQ), mental health disorders (CIDI-CAPI) and sociodemographic characteristics.

**Results:**

Of the 1754 adolescents interviewed 5.3% (95% CI 4.1 to 6.9) mentioned a change in residence due to violence. Among them 38.5% lived in poverty compared to 23.6% of those non-displaced by the conflict. Suicidal thoughts and suicide attempt were present in 19.8% and 9.1% of displaced adolescents respectively, compared to 5.8% and 2.1% of non-displaced adolescents. The prevalence of post-traumatic stress disorder (PTSD) and any mental health disorder (measured with the CIDI-CAPI) was higher in the displaced population 12.3%, 11% respectively, in contrast to 2.1% and 7% of those non-displaced. Finally, anxiety and depressive disorders were more common among displaced adolescents.

**Conclusion:**

A higher prevalence of mental health conditions and disorders is observed among displaced adolescents.

## Background

Colombia is considered a violent country, due to its long national history of internal displacement and high rates of interpersonal violence. [1, 2]. The Colombian internal armed conflict began around the 1950s, boomed in the early eighties and declined in 2018 [1] as a consequence of peace agreements [3]. Even though multiple strategies have been implemented to cope with the consequences of this situation, in 2017 Colombia was still the second country in the world with the highest number of displaced individuals, the majority due to armed conflict [4].

In 1985, Colombia established a legal framework to collect accurate information about the number of displaced individuals in the country. However, these early records were inaccurate. With the creation of the National Registry of Victims (*Registro Único de Víctimas*, RUV) in 2002, the reliability of the registered numbers improved [3]. The National Center of Historical Memory gives an account of the magnitude of violence the country experienced between 1958 and 2012 with an estimate of 220,000 deaths [5], 25,007 missing individuals, 1754 victims of sexual violence, 6421 children and adolescents recruited by armed groups and 4,744,046 displaced individuals. Additionally, it is estimated that between 1985 and 1995 around 819,510 were displaced due to the armed conflict, which suggests that, altogether around 5,700,000 people (12% of the Colombian population) have been displaced [3, 5].

Other international crises, like the one currently affecting Syria, have brought to light the consequences of violence on mental health [6]. Worldwide, there are 51 ongoing armed conflicts and approximately 65 million displaced people [7]. As Colombia’s case, most of the affected territories are low or middle income countries (LMIC) [8]. Colombia is one of the few places in the western hemisphere where conflict displacement persists [9]; nonetheless, the majority of information about mental health consequences comes from retrospective studies conducted in other affected countries [8]. Several studies demonstrate that individuals displaced by violence are at higher risk of mental health issues or diseases [10]. The most frequently made associations between mental diseases and conflict include post-traumatic stress disorder (PTSD) [8, 11], anxiety disorders, mood disorders and sleep disorders [11, 12].

Perhaps more concerning are the effects of these situations in children and adolescents [13], for whom other studies have demonstrated a higher susceptibility to mental health conditions [14]. Worldwide, half of the global displaced population are children and adolescents [11], nevertheless both populations have been poorly studied, which raises an ethical concern. Considering that adolescents who are exposed to social problems, such as discrimination or exclusion, tend to experience more emotional, adaptive and sanitary issues, internal displacement makes this age group particularly vulnerable [15]. In Colombia, 28% of individuals are 15 years old or less, and yet they represent 39% of displaced individuals [3]. Due to this situation of violence and displacement, in 2011 the Colombian government implemented a psychosocial, physical and mental support program for registered victims (*Programa de atención psicosocial y salud integral a* víctimas; PAPSIVI), which assesses individual and collective measures in order to assist adolescents victims of displacement [16]. However, the effects of this program are unknown as they have not yet been studied. Considering that adolescence is a period of great susceptibility for changes due to the undergoing biological and social transitions, as well as a period of huge importance for physical and mental welfare, the in-depth study of the impact of violent displacement on this vulnerable population is indispensable [17].

Additionally, previous studies have shown important socio-economic and health consequences on the displaced Colombian population [10, 14, 18]. Repercussions of the armed conflict on both individual and public health clearly demand an urgent intervention [19] and further studies that may retrieve useful information for the improvement of such interventions [20].

The present study therefore aims to describe the prevalence and associations of mental illnesses in Colombian adolescents exposed to violence, based on data from the fourth version of the Colombian National Mental Health Survey (NMHS) from 2015. By doing so, this study will contribute to understand the needs of this vulnerable population.

## Methods

### Population sample

Based on data from the NMHS 2015, we performed a secondary analysis of the sample of Colombian adolescents aged 12–17 years. The NMHS is a cross-sectional mental health survey applied to a sub-sample, retrieved from a larger national sample, and designed by the Colombian Ministry of Health and Social Protection. This sample has national and regional representability [21]. Therefore, our results are representative of the adolescent population from Colombia. For more details see the NMHS protocol [21].

## Measurement

### Identification of the individuals displaced by the conflict

To identify adolescents displaced by violence we used the following question: “Did any of the mentioned residence changes occur because your life or the life of someone living with you was threatened?”

### Sociodemographic variables

The variables of gender, age, schooling, educational level, region and urban or rural area were evaluated. Family dysfunction was evaluated with the family APGAR score [22] and poverty was assessed using the Global Multidimensional Poverty Index (MPI). The results of the latter were divided into five dimensions according to the number of deprivations each individual experienced [23].

### Mental health

Alcohol consumption was analyzed using the World Health Organization (WHO) measuring tool “Alcohol Use Disorders Identification Test” (AUDIT). Specifically, we performed a prior alcohol abuse screening with the AUDIT-C, which is a shorter version, when it was positive the full version was performed. This screening can help identify individuals with hazardous drinking habits or those who have active alcohol abuse or dependence [24].

To evaluate other mental illness diagnoses, we used the electronic version of the Composite International Diagnostic Interview (CIDI-CAPI 3.0). We studied the presence of any mental health disorder, including major and minor depressive disorders, dysthymia, mania, generalized anxiety disorder, panic disorder, social phobia and suicide ideation [25]. These conditions were also dichotomized into two main categories: anxiety disorders and affective disorders. To assess the presence of anxiety, depression, psychosis or epilepsy symptoms, we used the self-report questionnaire (SQR), also created by the WHO [26].

Finally, in order to evaluate post-traumatic stress disorder (PTSD) symptoms we used a modified version of the Post-Traumatic Stress Disorder Checklist (PCL-C) for civil population [27]. Given that the prevalence of PTSD was expected to be low, we applied the PCL-C only to individuals who had experienced at least one traumatic event during their lives. To consider a positive PCL-C, we followed Brewin’s recommendations [28].

All the instruments were chosen during the implementation of the NMHS. These were selected by experts according to their characteristics and uses.

## Statistical analysis

The percentage of individuals displaced by violence was estimated with its corresponding 95% confidence interval using the Taylor linearization series method to estimate variance in complex surveys. The association between the percentage of adolescents displaced by violence and their demographic characteristics and mental illnesses was evaluated using Pearson’s χ^2^ independent test, which was modified according to Rao and Scott’s second-order method, taking into account the survey´s polietapic design [29]. All analyses were performed in STATA 15.0.

## Results

### Displacement prevalence among adolescents

Of all the participants interviewed in the 2015 NMHS, 1754 were adolescents and 5.3% (95% CI 4.1 to 6.9) reported a change of residence due to violent threats.

### Sociodemographic characteristics

Table 1 shows the distribution of the demographic characteristics of displaced and non-displaced adolescents. In this table, a possible association between the variables of gender and schooling was observed. The displaced population showed a larger proportion of female adolescents who lack formal education.

**Table 1:** Demographic characteristic of the adolescent population

	Change of residence due to threats of violence				
	Yes		No		p*
	n	%	n	%	
Gender					
Male	40	36.5	807	50.2	*0.044*
Female	62	63.5	845	49.8	
Age					
12	10	5.6	264	17.5	*0.042*
13	21	21.2	297	16.7	
14	23	18.4	256	15.4	
15	12	10.5	287	17.2	
16	17	26.5	258	15.4	
17	19	17.8	290	17.9	
Schooling					
Yes	78	76.4	1426	86.9	*0.026*
No	24	23.6	226	13.1	
Education level					
None/Primary	72	70.9	1044	62.5	0.39
Secondary	30	29.1	604	37.3	
Technical	0	0.0	4	0.2	
Region					
Central	25	34.2	305	23.4	< *0.001*
Atlantic	12	10.6	400	24.4	
Bogota	14	6.7	286	14.9	
Oriental	21	17.8	390	21.1	
Pacific	30	30.7	271	16.3	
Area					
Urban	76	71.9	1286	74.1	0.71
Rural	26	28.1	366	25.9	
Poverty					
Not living in poverty	65	61.5	1303	76.4	*0.012*
Living in poverty	37	38.5	349	23.6	

* Bivariate analysis with Chi square test to compare displaced adolescents and non-displaced, significant at p < 0.05 (italics)

Moreover, we found a higher proportion of adolescents aged 13 and 16 in the displaced population compared to the non-displaced population. We also found differences regarding their socioeconomic status. Through the MPI measurement we saw that 38.5% of displaced individuals belonged to lower income backgrounds versus 23.6% of individuals who were not displaced by violence. Furthermore, results revealed that the regional distribution of these two populations across the nation were different. A larger proportion of displaced individuals was found in the Central and Pacific regions of the country.

### Mental health

Regarding the mental health characteristics seen in Table 2, displaced adolescent population frequently self-reported medium and high anxiety and depression symptoms in the SQR questionnaire. For both conditions and items, the percentage of individuals with positive results among displaced adolescents was almost twice the percentage of non-displaced adolescents with positive results.

**Table 2:** Evaluation of mental, neurobiological and behavioural disorders in the adolescent population

Change of residence due to threats of violence					
	Yes		No		p*
	n	%	n	%	
Any lifetime mental health disorder					
Yes	13	11.0	124	7.0	0.18
No	89	89.0	1528	93.0	
Any lifetime anxiety disorder					
Yes	9	8.8	87	4.8	0.13
No	93	91.2	1565	95.2	
Any lifetime affective disorder					
Yes	7	5.0	48	2.8	0.19
No	95	95.0	1604	97.2	
Anxiety SQR					
None	31	31.6	803	50.3	< *0.001*
Low number of anxiety symptoms (1–2)	38	35.4	603	35.4	
Medium number of anxiety symptoms (3–4)	22	23.5	171	10.1	
High number of anxiety symptoms (> 5)	11	9.5	75	4.2	
Depression SQR					
Low number of depression symptoms (1–3)	66	61.7	1337	80.8	*0.001*
Medium number of depression symptoms (4–6)	25	29.7	255	15.5	
High number of depression symptoms (> 7)	11	8.6	60	3.7	
Psychosis SQR					
None	81	77.3	1483	90.0	*0.002*
1 out of 2 symptoms indicating positive psychosis	21	22.7	169	10.0	
Epilepsy SQR					
None	96	94.7	1619	98.4	*0.009*
Suggestive symptom of positive epilepsy	6	5.3	33	1.6	
Global SQR					
Negative	76	71.6	1450	88.4	< *0.001*
Positive	26	28.4	202	11.6	
Suicidal thoughts					
Yes	17	19.8	71	5.8	< *0.001*
No	66	80.2	1241	94.2	
Suicidal plan					
Yes	3	5.8	20	1.5	0.1
No	80	94.2	1292	98.5	
Suicide attempt					
Yes	8	9.2	23	2.1	*0.008*
No	75	90.8	1289	97.9	
PTSD					
Yes	5	12.3	12	2.7	*0.007*
No	34	87.7	477	97.3	
AUDIT/alcohol					
Probable alcohol dependence	0	0.0	3	0.2	0.28
Hazardous drinker	5	4.7	44	2.1	
Excessive consumption	0	0.0	42	2.7	
None of the above	97	95.3	1563	95.0	
Family dysfunction					
None	84	85.4	1363	84.1	0.79
Slight family dysfunction	12	10.7	179	9.9	
Moderate family dysfunction	4	2.2	70	3.8	
Severe family dysfunction	2	1.6	40	2.2	

* Bivariate analysis with Chi square test to compare displaced adolescents and non-displaced, significant at p < 0.05 (italics)

A higher frequency of positive symptoms suggesting psychosis and epilepsy were reported by those violently displaced. For both conditions, the proportion of displaced individuals who presented symptoms was greater than twice the proportion of non-displaced with symptoms. Regarding suicide, which was evaluated using three items, only two had a statistically significant p-value (suicidal ideation and history of suicide attempt). Among displaced individuals, 19.8% revealed suicidal ideation, while 5.8% of the non-displaced had a positive answer. In the case of suicide attempt, 9.1% of the displaced population responded positively versus 2.1% who were not displaced. Additionally, having a potential PTSD was higher among the displaced population with 12.3% responding positively versus 2.1%.

## Discussion

In a historically violent national context, which affects individuals and their communities, it is important to study vulnerable populations in order to promote successful public health interventions. Colombia is one of the most violent countries across the world and the second in terms of conflict-displaced individuals [20]. Worldwide, half of the displaced population are children and adolescents [11]; however, Colombia lacks sufficient information regarding the consequences of the conflict in this age group. Moreover the information that does exist is hardly generalizable [30, 31]. Although some regulations have been implemented,the affected population continues to endure a state of vulnerability [32]. Furthermore, health access barriers continue to limit proper medical care for displaced adolescents.

Overall, we found associations between forced displacement and both sociodemographic characteristics (socioeconomic status, schooling, gender, region) and mental illnesses (depression, anxiety, psychosis, suicidal attempt, suicidal ideation, PTSD). These are the first results in a sample of adolescents nationally representative.

Low rates of education among displaced adolescents have been previously corroborated in other contexts in which an armed conflict was or is present. International reports related to violence associated with war report that most victims do not have an education or at most have only completed primary education [33]. However, a study analyzing the data of the 2015 NMHS in displaced children 11 years or younger, demonstrates that a higher proportion (98.7%) of children were schooled [14]. In a Colombian context, a study described that most children and adolescents displaced by violence, who presented mental health conditions, did not have any formal education [31]. Consequently, it would be relevant to investigate the barriers and factors related to school attendance between childhood and adolescence for those who are victims of displacement due to an armed conflict. The relevance of this result arises from the previously described association between a greater number of mental illnesses in the adolescent and adult population and a lower educational level. It is worth mentioning that individuals with low levels of education also have a greater risk of being exposed to traumatic events related to violence [17].

In Latin America individuals who have a basic or secondary education and have been exposed to traumatic violent events present higher proportions of mental illness, such as PTSD, compared to those with higher educational degrees [34]. Such is the importance of this last aspect, that emerging international organizations centered on violent contexts have highlighted that the access to education is vital in order to reach a state of well-being, particularly in female adolescents who have experienced vivid violent events related to armed conflict [17]. On the other hand, we observed that family dysfunction was similar for both displaced and non-displaced adolescents. This suggests that families may experience a greater cohesion following a traumatic event of displacement. However, this study only allows us to generate questions and future studies might generate new conclusions.

Our results show a larger proportion of female youth among the displaced individuals. This is consistent with other results for both adults and adolescents [9, 35, 36]. In the context of war there is, not only a larger number of female adolescents who have had contact with war, but also a greater prevalence of mental and psychological problems, such as insomnia, depressive symptoms, anxiety, irritability and low self-esteem, among others. These have been identified through questionnaires widely used worldwide [36]. A higher prevalence of women in these circumstances demands a quick response due to disadvantages they face, which are a result of social inequality within the Colombian context (i.e. abuse, economic difficulties, verbal and physical aggression). These can then translate into adverse health outcomes, both physical and mental [37]. Variables, such as low educational levels in female adolescents, have been associated with other public health problems such as early pregnancy [38] and a higher number of depressive and anxiety disorders [39]. Additionally, during an armed conflict rates of sexual violence tend to increase. Although sexual violence can rarely be perpetrated by women, most cases are performed by men towards women. Studies of this phenomenon highlight that this type of violence is perpetrated against individuals of any group of age specially when the victim is surrounded by an armed conflict [33], as it is the case of Colombia [32]. Poverty is frequently found among displaced populations, which is also described worldwide [17, 33, 36]. This factor was mentioned in different populations of adolescent women who were affected by armed conflict or displacement [40]. Additionally, it has been observed that sexual abuse does not only increase during an armed conflict, but also through the simple act of labelling these individuals as refugees. This could lead to more teenage pregnancies, which in the literature has shown increased risk of abuse and violence in the progeny [38], revealing a possible perpetuation of harmful factors in the wellbeing and health of victims of violence.

In addition to sociodemographic factors, aspects related to mental health were also recorded. This has been particularly studied due to the Syrian crisis. The results illustrate an important relationship between armed conflict, forced displacement and negative resilience with mental health conditions [35, 41]. In our study, 11% of all individuals had at least one mental health disorder. This result is consistent with those obtained for an adult sample, which was forced to migrate during their adolescence due to violence [35]. Among the statistically significant differences with the non-displaced population are symptoms of depression and anxiety. Both types of symptoms were found to be the most frequent according to a systematic review that assessed victims of sexual violence in the context of an armed conflict [33]. Taking this into account would be coherent for conducting an in-depth investigation for the situation of this population, especially considering that the majority of those affected are women [42]. Besides depression and anxiety, we also found a larger prevalence of suicidal thoughts in displaced adolescents. A previous study showed no difference in suicidal ideation in the displaced adult population [12]. Keeping in mind that adolescents are a more vulnerable age group, the lack of contingency strategies could be the reason that explains the difference within these two populations. This data highlights the need to establish programs to prevent the increase in suicide rate among this group, which not only has a higher rate of suicide ideation and attempts, but also shows a higher prevalence of mental problems and social vulnerability. Finally, a higher proportion of adolescents with PTSD is observed and this result repeats itself in other similar studies [43].

Regarding the mental health assistance provided to this population in Colombia, these results demonstrate the need for developing a stronger and more empirical PAPSIVI. This program should be consistent with the results of this study and allow both clinicians and health personnel, who treat adolescents, to implement interventions more efficiently. It is a priority to have preventative interventions. Religious activities [36], sports [40] and education [44] in similar affected populations have been studied with promising results. It is urgent to demonstrate this data in order to develop and implement early interventions and improve individual’s future quality of life.

### Limitations

Our study was a cross-sectional study; therefore, it could not evaluate causality nor temporality. Associations between the outcomes and forced displacement are described but not as causal relations. Additionally, given that the tools we used mainly depend on the recollection of the individuals, they could have led to certain biases. In general, the tools we used were validated and broadly accepted; nevertheless, the answers given only suggest the presence of conditions and not a particular diagnosis, which diminishes accuracy. To identify PTSD we used a modified version of the PCL-C and we only asked individuals who had experienced one traumatic event; therefore, the prevalence found may be higher than the real one. Moreover, it is not comparable to the prevalence of other conditions found using the CIDI-CAPI.

### Strengths

To our knowledge, this is the first study that describes these associations in a representative sample of the Colombian adolescent population. Even though the study is cross-sectional, the design was performed rigorously in collaboration with the Colombian Ministry of Health and Social Protection. Moreover, unlike other studies, our current study investigated displacement due to violence not only at a regional level, but also at a personal level. Not to mention, we were able to compare this group with a non-displaced one.

## Conclusion

The present study has a great representation at a national level because it is derived from the 2015 NMHS. It is also one of the first studies to analyze the displaced adolescent population. The results reveal a vulnerable population in a country with political conflicts, which has an important significance. In order to create coherent and beneficial interventions, which will contribute to displaced adolescent´s mental and physical needs, it is important to recognize that these circumstance bring with them repercussions at a sanitary level.

The studied age group and, perhaps even their caretakers, require interventions aiming to improve their mental and physical health. Displaced adolescents who are under the care of individuals with similar circumstances, may have more difficulties generating protective shield effects which could mean a larger number of physical and mental disorders. Therefore, our results should promote strategies which offer services to these patients in a collaborative care context.

## Data Availability

The datasets analyzed during the current study are available from the corresponding author on reasonable request.
